# *Sonerila nairii* (Melastomataceae) – a new species from the southern Western Ghats, India

**DOI:** 10.3897/phytokeys.62.7623

**Published:** 2016-03-25

**Authors:** Soumya Murugan, Maya C. Nair

**Affiliations:** 1 Environmental Resources Research Centre, Peroorkkada, Thiruvananthapuram, Kerala, PIN 695005, Indias; 2 Post Graduate and Research Department of Botany, Govt. Victoria College (University of Calicut), Palakkad, Kerala, PIN 678001, India

**Keywords:** *Sonerila*, Kerala, Nelliampathy, Palakkad, Western Ghats, India, critically endangered

## Abstract

The new species *Sonerila nairii* (Melastomataceae) is here described from Pothumala of the Nelliampathy hill ranges of Western Ghats of Kerala, India. Morphologically it most closely resembles *Sonerila erecta* and *Sonerila pulneyensis* from which differs by the form of the stem, leaves, peduncle, pedicel, inflorescence, pubescence of the stem, leaves and hypanthium, and by the form of stamens and stigma.

## Introduction

The genus *Sonerila* Roxb. (Melastomataceae) consists of erect or creeping and rhizomatous terrestrial low epiphytic herbs or semi-woody shrubs, occasionally acaulescent with a distribution in tropical Asia (Clausing and Renner 2001). The genus is classified under the tribe Sonerileae (Triana 1866) and is clearly delineated from other genera in having trimerous flowers and mostly uniparous scorpioid cymes (Sunil et al. 2014).

In Hooker’s Flora of British India, Clarke (1879) recognised 43 species and Gamble (1919) recognised 13 species of *Sonerila*. Lundin (1998) made an extensive documentation of Melastomataceae with special emphasis on *Sonerila* of South India. Lundin and Nordenstam (2009) considered the genus to have about 175 species distributed from Sri Lanka and India to the Indo-Pacific. As per recent assessment by considering the works published after Gamble’s 1919 treatment, the genus is represented by 52 species in India and Western Ghats has the highest species diversity with about 35 species (Nayar 1976, Giri and Nayar 1985, 1986a,b, 1987, Prakash and Mehrotra 1988, Gopalan and Henry 1989, Giri et al. 1992, Ravikumar 1999, Murugan and Manickam 2002, Josephine et al. 2003, Lundin and Nordenstam 2009, Murugesan and Balasubramaniam 2011, Narayanan et al. 2013, 2014a, b, Deepthikumary and Pandurangan 2014, Sunil et al. 2014, Narayanan et al. 2015).

During the field exploration in the Kollengode range of Nemmara forest division, an interesting *Sonerila* was collected from the Pothumala region of Nelliampathy hills of Palakkad district during October 2015, at an altitude of about 1140–1160 m. Critical analysis of the specimen and comparison with protologues and digital images of herbarium specimens of the closely allied species, *Sonerila erecta* Jack (Barcode id: K000867797) and *Sonerila pulneyensis* Gamble (Barcode id: K00867655) deposited at Kew Herbarium revealed its distinctiveness from these and other allied species. Therefore this taxon is here described as the new species *Sonerila nairii*.

## Description of the new species

### Sonerila nairii

Taxon classificationPlantaeMyrtalesMelastomataceae

Soumya & Maya sp. nov.

urn:lsid:ipni.org:names:77153915-1

Figs 1 , 2

#### Diagnosis.

The new species is distinguished from *Sonerila erecta* and *Sonerila pulneyensis* by the decumbent unbranched stem, absence of a distinct peduncle, the cymose 1–2 flowered terminal inflorescence and by the anthers which are half the length of filament. (*Sonerila erecta* and *Sonerila pulneyensis* have branched stem, a distinct peduncle, inflorescence consisting of more than 2 flowers in a cyme and anther having same the length of the filament.)

#### Type.

INDIA. Kerala: Palakkad district, Pothumala, Nelliampathy hills, 10°30'09.6"N; 76°42'16.5"E, 1160 m 18 Oct 2015, *Soumya M. & Maya C. Nair 1185* (Holotype CALI!, isotypes MH!, ERRCH!, GVCH!)

#### Description.

Decumbent, unbranched, delicate, succulent herbs attaining 6–10 cm height; the lower portion more or less trailing and bear perennating buds, while the upper portion curves upward and grows erectly. Stem translucent, fleshy, subterete with scattered multicellular and glandular trichomes which form a dense hairy nodal ring. Leaves opposite, fleshy, petiole 0.5–1.5 cm, adaxially grooved, with glandular trichomes; lamina ovate, 1.3–2.5 × 1–1.5 cm, base obtuse, green with pink tinge below, upper surface densely hirsute (0.08–0.09 × 0.03–0.06 cm), lower surface with scattered glandular trichomes (0.02–0.03 cm × 0.03–0.04 cm), margins finely serrate, acute at apex, prominently 3-nerved a pair of nerve obscurely seen near the margin. Inflorescence terminal, unbranched, condensed, a 1–2- flowered cyme. Peduncle more or less absent. Flowers 3-merous, pedicel 0.5–0.7 cm with few glandular trichomes, shorter than hypanthium, light green. Hypanthium 0.8–0.9 cm long, campanulate with scattered glandular trichomes, light green. Calyx lobes 3, 0.15–0.2 cm long, triangular, non-caducous, with sporadic glandular trichomes and pink tinge. Petals 3, 0.8–0.7 × 0.5 cm–0.45 cm orbicular-obovate, acuminate at apex with 3–4 glandular trichomes on the midrib of the abaxial side. Stamens 3; filaments 0.4–0.42 cm long, glabrous, white; anthers yellow, 0.2–0.22 cm, cordate at base, glabrous. Style 0.8–0.9 cm long, curved, deep pink towards the tip, stigma capitate, glabrous. Capsule campanulate, 0.8–0.9 cm long and 0.45 cm wide with occasional glandular trichomes, green. Seeds many, 0.07× 0.02 cm, minutely tuberculed, brown.

#### Phenology.

October–December.

#### Etymology.

The specific epithet honours Dr. P.K.K. Nair (1930-), eminent scientist, renowned as father of Indian palynology and founder director of the Environmental Resources Research Centre (ERRC), Thiruvananthapuram.

#### Distribution and ecology.

*Sonerila nairii* grows at altitudes of 1140–1160 m in shady rock surfaces within moist loose soils and under the evergreen canopies along the Pothumala hill tract of Nelliampathy hills. In these habitats, *Sonerila nairii* grows close association with crustose lichens. The new species seems to prefer more or less moist lithophytic habitats in contrast to *Sonerila erecta* and *Sonerila pulneyensis* which grow in evergreen and riparian forests respectively. The distribution of the three taxa has been summarized in Fig. 3.

**Figure 1.**
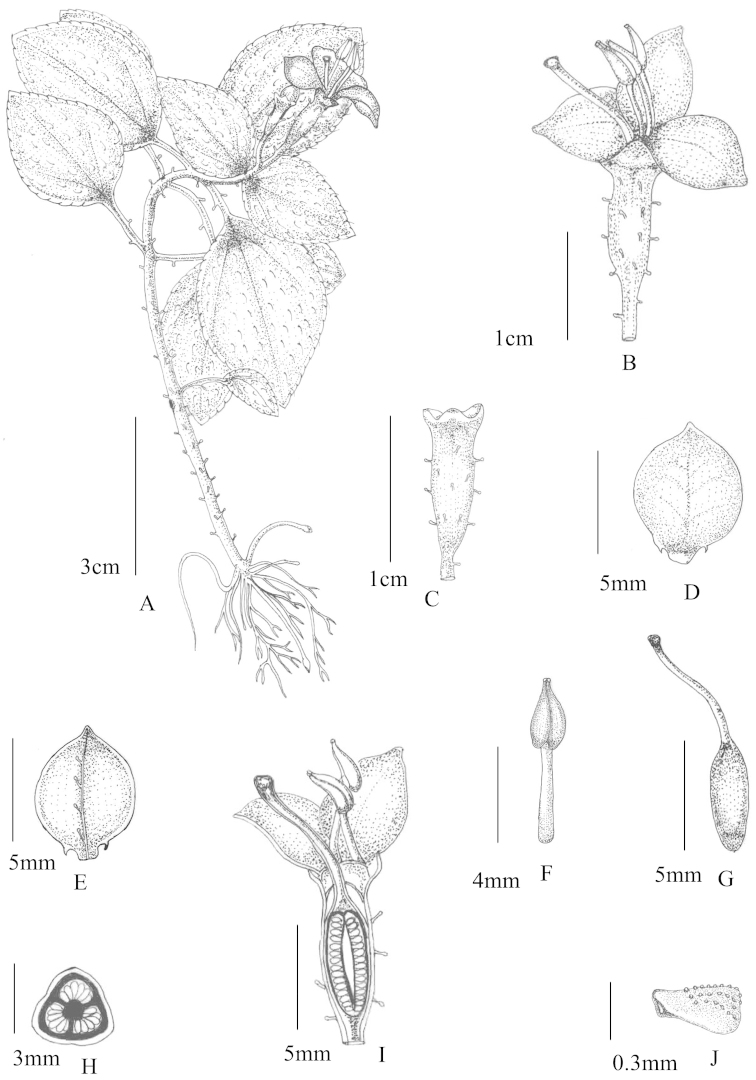
*Sonerila nairii* **A** Habit **B** Single flower and flower bud **C** Calyx **D** Petal-Adaxial side **E** Petal-Abaxial side **F** Stamen **G** Gynoecium **H** Ovary TS **I** Flower LS **J** Seed (from *Soumya. M & Maya. C. Nair 1185*) Illustration by Soumya. M.

**Figure 2.**
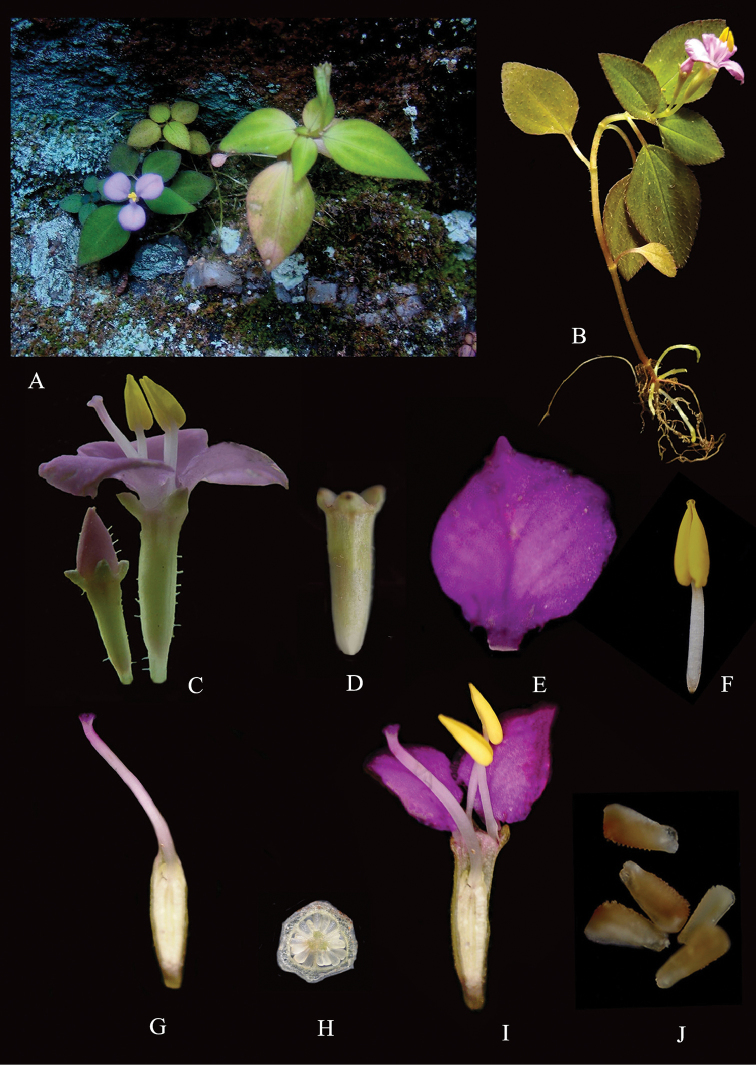
*Sonerila nairii* **A** Habitat **B** Habit **C** Single flower and flower bud **D** Calyx **E** Petal-Adaxial side **F** Stamen **G** Gynoecium **H** Ovary TS **I** Flower LS **J** Seed (from *Soumya. M & Maya. C. Nair 1185*) Photos by Soumya. M & Maya. C. Nair.

**Figure 3.**
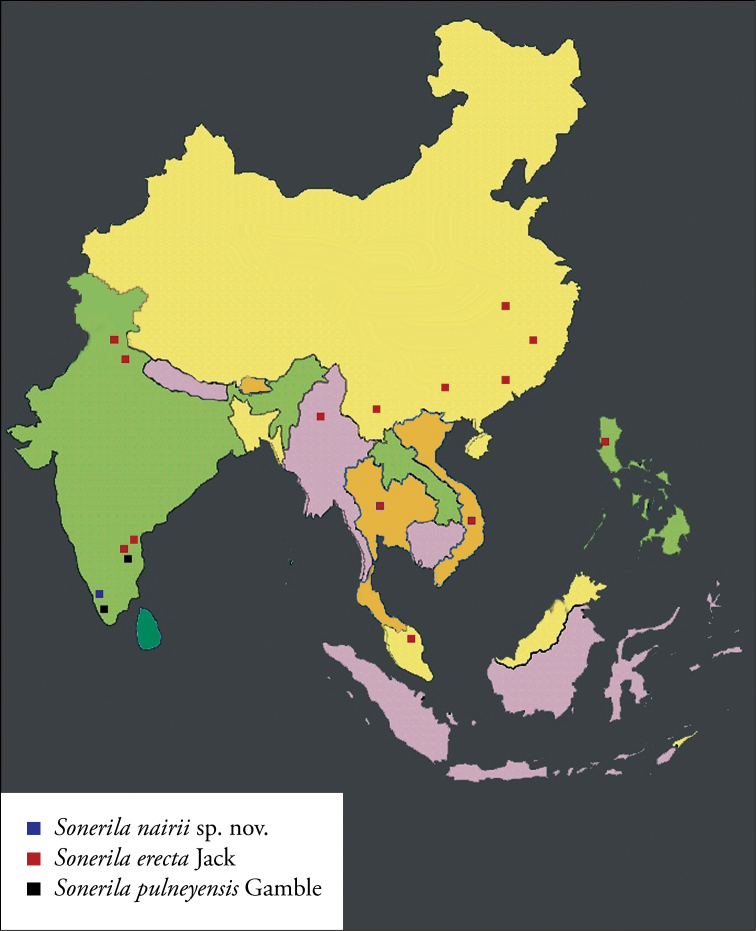
Map showing the distribution of *Sonerila nairii* sp. nov., *Sonerila erecta* Jack and *Sonerila pulneyensis* Gamble.

#### Conservation status.

Two populations comprising only a few individuals (5–10) of the species were recorded growing within a distance (50 meters) of each other. Apart from the type locality, the species has yet to be found anywhere else. Because the number of mature individuals is less than 50 and the species has a very restricted area of occupancy, we assign the species, the status of Critically endangered using IUCN Strategies and criteria (IUCN 2014).

#### Additional specimens examined

**(Paratypes).** INDIA. Kerala: Palakkad district, Pothumala, Nelliampathy hills, 1 Nov 2015 *Soumya M. & Maya C. Nair 1187* (ERRCH!) (Environmental Resources Research Centre Herbarium), 5 Nov 2015 *Maya C. Nair & Soumya M. 98* (GVCH!) 12 Nov 2015 *Maya C. Nair & Soumya M. 99* (GVCH!) (Government Victoria College Herbarium)

#### Discussion.

*Sonerila nairii* differs from *Sonerila erecta* by having an unbranched decumbent stem, leaf margins with pink tinge; by the absence of a peduncle, a condensed, cymose, 1–2-flowered, terminal inflorescence. The anthers are half the length of the filaments and the stigma is capitate. From *Sonerila pulneyensis* Gamble it differs in having decumbent and sparse glandular trichomes on the stem and pedicel, dimorphic hairs on the leaves, by the absence of a distinct peduncle; by the terminal inflorescence of 1-2 flowers borne in a condensed cyme. The hypanthium is green-coloured and the anthers are half the length of the filaments. Further differences between *Sonerila nairii*, *Sonerila erecta* and *Sonerila pulneyensis* are given in Table 1.

**Table 1.** Taxonomic delineation of *Sonerila nairii* from *Sonerila erecta* and *Sonerila pulneyensis*.

Taxonomic traits	*Sonerila nairii* sp. nov.	*Sonerila erecta*	*Sonerila pulneyensis*
Habitat	Shady rocks within evergreen forest	Evergreen forests	Riparian forests in high altitudes
Stem	Decumbent, sub-terete succulent, unbranched with sparse glandular trichomes	Erect, 4-angled, slender, branched with sparse glandular trichomes	Creeping, sub-succulent, branched, glabrous
Nodal region	Glandular trichomes arranged in a nodal ring	Not prominent	Absent
Leaf	Petiole 0.5–1.5 cm long, angular, sparsely with glandular hairs; lamina slightly coriaceous, ovate, 1.3–2.5 × 1 –1.5 cm, prominently 3-nerved, an additional pair of nerve obscurely seen near the margin	Petiole 0.4–1.5 cm long; lamina membraneous, narrowly elliptical to ovate, 1–2.5 × 0.4–1.6 cm, Secondary veins 2–3 pairs	Petiole 0.5–1.5 cm; lamina coriaceous, ovate 1- nerved, 2–5 × 1–3 cm
Nature of hairs on leaf lamina	Dimorphic hairs present, densely hirsute above and with sparse glandular trichomes below	Dimorphic hairs present, densely hirsute above and with sparse glandular trichomes below	Absent
Leaf margin	Narrowly serrate with pink tinge	Serrate without pink tinge	Broadly serrate with pink tinge
Postion of Inflorescence	Terminal	Terminal	Axillary or terminal
Inflorescence	Usually 1-2 flowered compressed terminal cyme	Inflorescences occurs at the end of branches, in 1-5 (up to 11-flowered) terminal scorpioid cymes.	Axillary or terminal 5- flowered umbellate cyme
Peduncle	More or less absent	Up to 2cm, with sparse glandular hairs	3–4cm long, glabrous
Pedicel	Pedicel 0.5–0.7cm with sparse glandular trichomes shorter than hypanthium	Pedicel 0.2–0.7cm with sparse glandular trichomes, shorter than hypanthium	Pedicel 1cm long, glabrous, length equalling hypanthium
Hypanthium	Light green with sparse glandular trichomes	Green with sparse glandular trichomes	Pink, glabrous
Petals	Petals pink to purple, broadly ovate, slightly clawed on either side,4–5 glandular trichomes on the mid rib below, tip acuminate	Petals pink to purple oblong-elliptic sparse glandular trichomes on the mid rib below, tip acute to acuminate	Petals rose, elliptic, glabrous,tip apiculate.
Stamen	Anthers half the length of filaments	Anthers as long as filaments	Anthers as long as filaments
Stigma	Capitate	Three lobed	Capitate
Capsule	Tubular, 3-sided with sparse glandular trichomes	Tubular, 3-sided with sparse glandular trichomes	Campanulate, glabrous

## Supplementary Material

XML Treatment for Sonerila nairii
